# Technical Pearls to Effectively Use 5-ALA in Fluorescence-Guided Tumor Resection—5 Lessons from the Operating Room

**DOI:** 10.3390/brainsci13030411

**Published:** 2023-02-27

**Authors:** Giuseppe Maria Della Pepa, Grazia Menna, Alessandro Olivi

**Affiliations:** Institute of Neurosurgery, Fondazione Policlinico Universitario Agostino Gemelli IRCCS, Catholic University, 00168 Rome, Italy

**Keywords:** 5-ALA, gliomas, surgery, education

## Abstract

Background: Since its introduction in 2007 in Europe and in 2017 in the United States, 5-ALA has demonstrated an undisputed advantage in providing real-time tumor visualization. The aim of the present paper is to summarize our institutional experience over a decade of routine 5-ALA-guided procedures in order to provide five surgical tricks to ease surgical workflow. Methods: Data were collected from 822 patients diagnosed with histopathologically confirmed high-grade gliomas (HGG)—according to the WHO 2021 criteria—who underwent surgery at the Fondazione Policlinico Universitario Agostino Gemelli between January 2012 and January 2022. Results: From our large institutional experience, the learned technical pearls were grouped in five distinct domains: 1. Analysis of visualization, overall workflow, and technical recommendations to improve intraoperative set-up; 2. Techniques to reduce the risk of inadvertent residuals and failure to evocate fluorescence; 3. Analysis of specific surgical conditions favoring remnants; 4. Assessment of different degrees of fluorescence and their surgical meaning; 5. Analysis of false positive cases. Conclusions: With all the limitations of a qualitative and retrospective analysis, this paper was specifically conceived as a vademecum for educational purposes to promote and maximize 5-ALA employment.

## 1. Introduction

Since its introduction in 2007 in Europe and in 2017 in the United States, the role of 5-ALA in improving the extent of rection (EOR) resection in high-grade gliomas (HGG) surgery has been pointed out by several qualitative analyses [1]. Evidence suggests that 5-ALA is the only available metabolic tumor-specific marker and provides real-time tumor visualization with minimal variations from the standard operative workflow [2,3,4,5]. Combining 5-ALA with functional mapping techniques and other intraoperative tools such as ultrasonography, surgeons can push the limits of surgical resection, preserving patients’ functional integrity [6].

Despite the pros, 5-ALA is not the “perfect” dye: first, it is best observed in a rather dark background, where anatomy and surgical instruments are barely visible; second, it could be easily hindered by cottonoid, cerebro-spinal fluid, or blood; third, fluorescence can be undermined by the presence of so-called “dark corridors”. The aim of the present paper is to summarize our institutional experience over a decade of 5-ALA-guided procedures. Other significant series have already confirmed 5-ALA to be associated with a greater EOR, with detailed description of factors limiting its accuracy and sensitivity and explaining how it can be combined with photodynamic therapy (PDT) [7,8,9]. However, this is the first paper explicitly dealing with operative nuances and specifically designed for teaching purposes.

## 2. Materials and Methods

Surgical data were collected from patients diagnosed with histopathologically confirmed HGG (according to the WHO 2021 criteria) who underwent surgery at the Fondazione Policlinico Universitario Agostino Gemelli between January 2012 and January 2022. A brain 1.5T MRI was performed as a conventional preoperative study in all cases. A contrast T1-weighted MRI sequence in combination with a Stealth Station Surgical Navigation System (Medtronic, Inc., Minneapolis, MI, USA) was used for preoperative planning and intraoperative neuronavigation. Technical suggestions to maximize 5-ALA effectiveness were collected from surgeons experienced in fluorescence-guided procedures. 

### 2.1. Operative Set-Up

Five-ALA (Medac GmbH, Wedel, Germany) was administered 4 to 6 h prior to surgery at a dose of 20 mg/kg body weight per os. Leica M720 OH5 (Leica Microsystems, Wetzlar, Germany) microscope was routinely used. Intraoperatively, 5-ALA regions of interest were defined under violet–blue illumination (Blue 400 filters). Electrophysiological monitoring (Nicolet Endeavor CR, Cardinal Health, Dublin, Ireland) and intraoperative neuronavigation were used in all cases. According to the surgeon’s request, CUSA (Integra Life Sciences, Princeton, NJ, USA) was employed. 

### 2.2. Ethics

Ethical approval was waived by the local ethical committee due to the retrospective nature of this study. All procedures involving human participants were performed in accordance with the ethical standards of the institutional and national research committee and the 1964 Helsinki Declaration and its later amendments. Informed consent was obtained from all individual participants included.

## 3. Results

**Figure 1:**
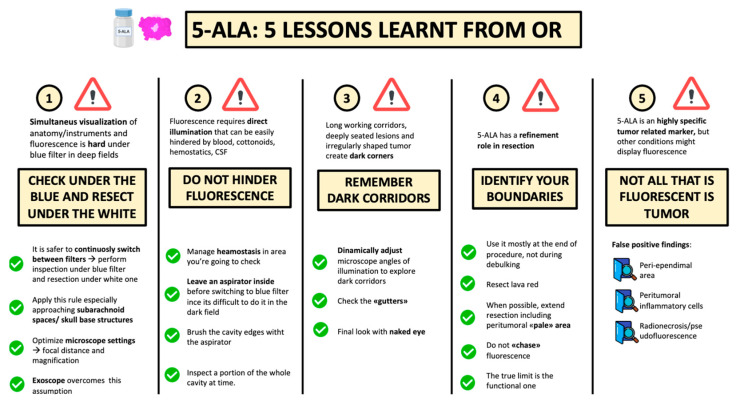
Pictorial summary of our findings.

A total of 822 cases of HGG underwent surgery at our institution in the examined period. Technical suggestions derived from our large institutional experience were grouped in five distinct domains:

(1) Analysis of the surgical workflow under 5-ALA;
(2) Ensemble of techniques to reduce the risk of inadvertent residuals and avoid false negatives;
(3) Consideration of specific conditions favoring remnants;
(4) Assessment of different degrees of fluorescence and their surgical and oncological meaning;
(5) Summary of false positive cases. (Figure 1)

## 4. Discussion 

### 4.1. “Check under Blue and Resect under White” (Unless You Have an Exoscope!)

As has been widely described, 5-ALA is a non-fluorescent prodrug, which is first absorbed by tumoral cells and then converted into a fluorescent protoporphyrin IX (PpIX). As a photosensitizer, PpIX is excited when placed under violet–blue light (370–440 nm), which is emitted by a specific filter added to the surgical microscope; tumor cells return red light in visible spectrum frequencies accordingly. To proceed with proper 5-ALA assessment, a second ultraviolet filter is added to the optical microscope lens [6]. Intraoperative visualization under blue filter is generally dark, while the tumor is characterized by a “lava red” appearance. Simultaneous visualization of both fluorescence and anatomy is possible only at the most superficial stages of resection. In the deepest stages, where illumination is poor and the surgical cavity’s edges further reduce illumination, anatomy is hardly distinguishable: vessels, brain parenchyma, nerves, and even dura can be evanescent [7,10]. Against this background, 5-ALA-guided resection should involve two phases continuously alternating during surgery: blue filter should be used for identification purposes, since it has a low degree of resolution, while white light should be used for proper resection. The microscope setting is paramount: under blue filter, maximal resolution occurs when the focal distance is adequate (generally below 350 mm) and at lower magnification (below 4× or more), as higher zoom results in suboptimal light conditions. The continuous switching between blue filter and white light to visualize fluoresce and anatomy, respectively, could be summed up as: “check under blue and resect under white”. Such a paradigm is true, especially when approaching subarachnoid spaces or the skull base, where the risk of inadvertent damage to vascular or nervous structures is more relevant. 

**Figure 2:**
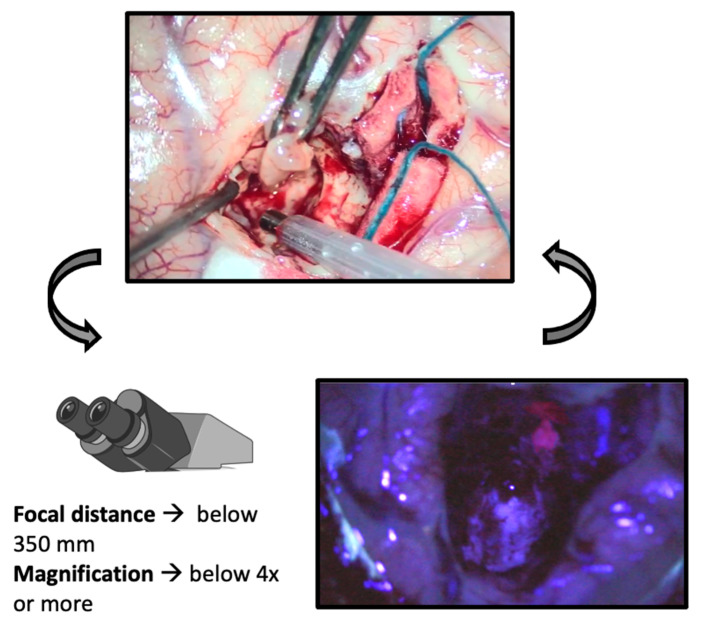
The figure embodies the principles of “check under blue” (**bottom right**), using an optimal microscope setting (**bottom left**), and “resect under white” (**top**). To achieve a satisfying resection, it is necessary to continuously switch from blue to white filter.

This paradigm changes only when using an exoscope instead of a surgical microscope. With the latter, fluorescence is captured under blue filter and transferred via digital video chip technology, and processed and projected via an optimized data transfer link, on high-resolution monitors [9]. This mechanism allows for high-quality image capture under very low light conditions, easing the simultaneous visualization of fluorescence and anatomy and enabling the surgeon to proceed with resection directly under blue light, even in cases of deep-seated lesions, potentially modifying the classical surgical workflow (Figure 2) [11,12].

### 4.2. Do Not Hinder Fluorescence

A tumor covered by blood, cottonoid, and overlapping normal brain, or even by CSF, will not show fluorescence under blue-light filter. This is of great significance when the microscope light fails to thoroughly illuminate the surgical field, such as in deep fields or in cases of “non-orthogonal working corridors”. Analogously, blood clots might hinder the presence of tumor remnants. To overcome these obstacles, at least three considerations are worth making. 

**Figure 3:**
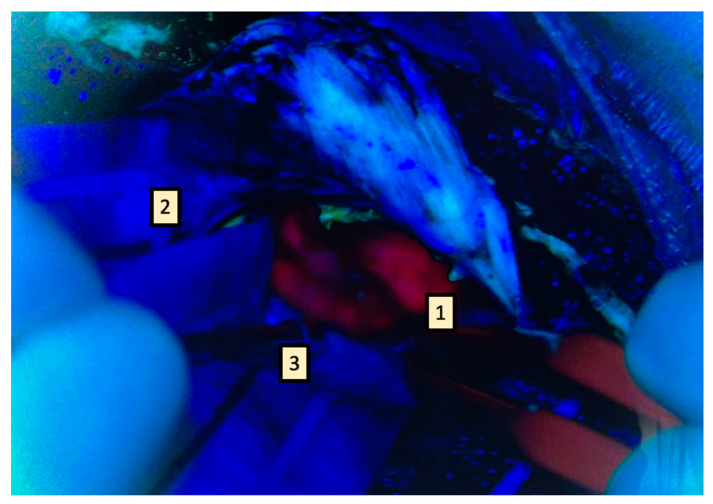
How to avoid hindering fluorescence. First, one portion of the cavity is inspected at a time (1) while the remaining part is covered with cottonoids (2). Hemostasis is performed in the limited (uncovered) area while cottonoids prevent bleeding from the rest of the cavity. The aspirator is introduced before switching to blue filter (this could be difficult to place in dark conditions), which is placed below the area being inspected (3). This keeps the cavity clean and allows the use of the aspirator to brush resection walls.

First, hemostasis should be carefully managed before checking the surgical cavity edges; hemostatics can hinder fluorescence, therefore they should be removed before inspection. Second, an aspirator should always be left in a safe position over a cottonoid, close to the area of inspection and before switching to blue filter. It is crucial to leave it inside the surgical cavity, otherwise it might be difficult to introduce it in a rather dark environment. Providing suction, the aspirator keeps the field clean during inspection and could be used to “brush” the area of interest, thus maximizing fluorescence visualization. Third, it is helpful to inspect one portion of the cavity at a time and cover the rest with cottonoids; this allows to focus microscope light efficiently rapidly perform a temporary haemo-stasis on a limited area (while the rest of it is controlled by cottonoids) and take ad-vantage of aspiration as explained before. (Figure 3).

### 4.3. Remember Dark Corridors

When working with 5-ALA, it is paramount to be aware of conditions that could impair fluorescence.

In the case of deep working corridors, microscope light might fail to thoroughly illuminate the surgical field, resulting in “dark corners”. This word stands for those deeply seated lesions or irregular, multi-lobulated tumors whose shape might impair 5-ALA fluorescence as the resection proceeds [13]. The issue of dark corridors is also true when speaking of non-exposed nodules, covered by a layer of normal tissue, or when the direction of resection changes by exploiting non-orthogonal planes. This also happens—regardless of tumor characteristics— in the latest stages of resection, since overlying brain edges might cover up residuals resulting in the so-called “gutter effect”. Tumoral edges tend to collapse within the surgical cavity, creating shadowing and resulting in a failure to identify pathological fluorescence. 

**Figure 4:**
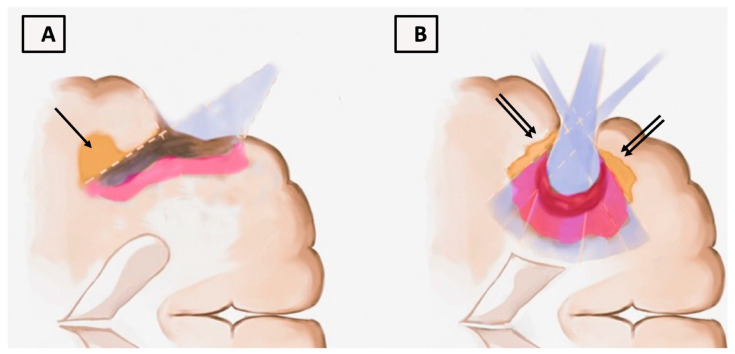
Long working corridors, deeply seated or irregularly shaped lesions, and surgical cavity edges can create dark corridors not allowing the microscope to elicit fluorescence from pathological tissue. (**A**) Shows the difficulties in identifying the remnants on the top left of the resection cavity (arrow). (**B**) During the final inspection, the microscope should be angled to extreme positions (double arrows) to achieve a complete exploration of surgical cavity edges.

A meticulous final inspection, dynamically adjusting the microscope’s working angles to extreme positions, is therefore necessary. Furthermore, after completing resection and hemostasis, and before SurgiSeal is placed in the cavity, the microscope with the blue filter on should be moved away from the surgical field to allow a final look with the naked eye. In our experience, this offers a better overview of the surgical field and helps to identify inadvertent remnants (Figure 4).

### 4.4. Identify Your Boundaries

Since experienced surgeons easily identify the core pathological tissue, 5-ALA has a mainly refinement role rather than being the pillar on which to build resection [14]. 

**Figure 5:**
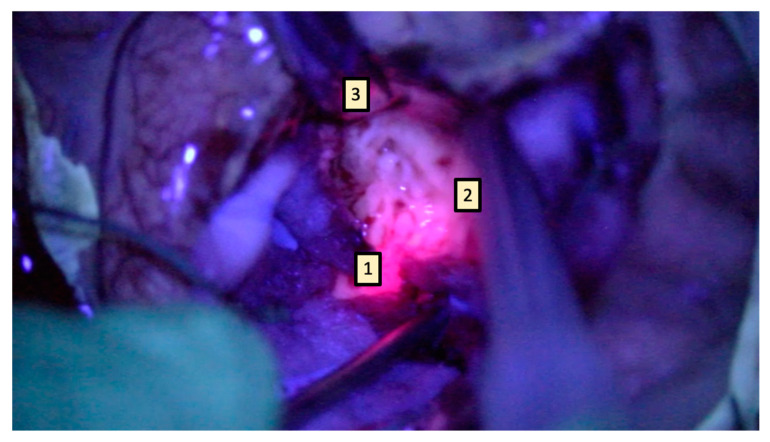
Research suggests that 5-ALA has mainly a refinement role after gross resection. The lava red area represents the tumor core (1) and the pale area represents the infiltrative margins (2), surrounded by the negative fluorescent area (3). Where feasible, it is paramount to extend resection, including the peritumoral “pale” area, without pushing it over neurophysiological boundaries.

It is known that the core of the tumor displays a clear “lava red” appearance, generally corresponding to gadolinium enhancement, while surrounding tissue displays a “pale” fluorescence. This “pale” area, extending over the limits of radiological enhancement, is a fertile soil for pathological infiltration. Where feasible, resection should also include these zones [15,16,17]. In other words, 5-ALA guidance is paramount in the final stages of surgery, whereas most of the procedure should be carried out under normal white light. However, especially with surgeons who are not routinely experienced with 5-ALA use, this might lead to a greedy approach, “chasing” fluorescence. This means that the surgical cavity should be carefully explored with blue filter only at the end of gross total resection in order to identify remnants to be resected if functionally feasible. It should always be kept in mind that functional integrity represents the true limit of resection and the procedure, especially in these final stages, should be supplemented by neurophysiological subcortical stimulation if necessary [18] (Figure 5).

### 4.5. Not All That Is Fluorescent Is Tumor

Evidence suggests that 5-ALA is known to be the only reliable tumor-specific marker, as widely demonstrated by previous experience [5]. 

Nevertheless, normal anatomical structures can also display some degree of fluorescence. This should be kept in mind when working in proximity to ventricular edges: the periependymal area shows a physiological “pale” fluorescence which should be distinguished from the “pale” fluorescence of the tumoral periphery. 

In addition, false positive findings can result due to the presence of peritumoral inflammatory cells (especially macrophages) and/or perifocal edema. Regarding the first, histiocytes/macrophages and lymphocytes can internalize 5-ALA: this may lead to a significant build-up of porphyrin precursors, making physiological tissue fluorescent. About the latter, 5-ALA could leak and accumulate extracellularly in lesions with a disrupted blood–brain barrier, such as in the case of pronounced perifocal edema. Furthermore, strong 5-ALA uptake can be observed in the specific setting of radionecrosis/late effects of chemo–radiotherapy treatment. 

**Figure 6:**
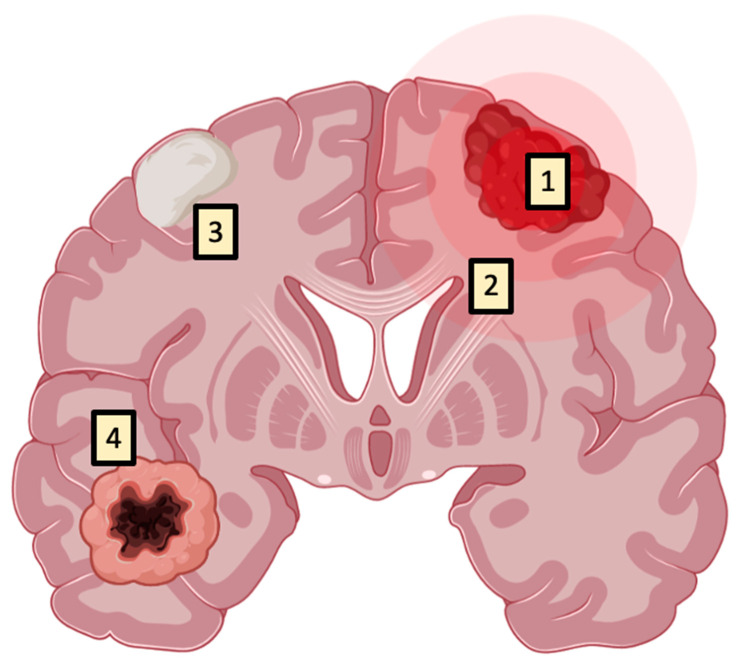
Even if 5-ALA is a highly tumor-specific tracer (1), fluorescence could be observed in normal anatomical structures such as the periventricular area (2) or in conditions or systemic inflammatory pathologies such as multiple sclerosis, brain abscesses (3), and other inflammatory diseases involving the brain, or in cases of radionecrosis (4) due to the presence of inflammatory infiltrates.

Fluorescence has also been observed in non-oncological settings, such as in multiple sclerosis, neurodegenerative diseases, and infectious conditions. Notably, all of these conditions share a conspicuous number of immune cells which, as mentioned before, can display fluorescence due to 5-ALA uptake and metabolism. In addition, the macroscopic fluorescence in bacterial brain abscesses could be explained by the bacteria capability to elaborate porphyrin precursors, since they are useful cofactors for their growth [19,20,21] (Figure 6).

## 5. Conclusions

With all the limitations of a qualitative and retrospective analysis, the present paper summarizes our institutional experience over a decade of routine 5-ALA-guided procedures in brain tumor surgery. It was specifically conceived as a vademecum for educational purposes. In our opinion, the reported technical strategies could and should be applied to promote and maximize 5-ALA employment in people undergoing surgery for HGG.

## Data Availability

Not applicable.
